# Treatment of Long-standing Condylar Dislocation with Vertical Ramus Osteotomy: A Case Report

**DOI:** 10.15171/joddd.2015.011

**Published:** 2015-03-04

**Authors:** Saeed Nezafati, Leila Ashkhasi, Mohammadali Ghavimi

**Affiliations:** ^1^Associate Professor, Department of Oral and Maxillofacial Surgery, Faculty of Dentistry, Tabriz University of Medical Sciences, Tabriz, Iran; ^2^Private Practice, Tabriz, Iran; ^3^Assistant Professor, Department of Oral and Maxillofacial Surgery, Faculty of Dentistry, Tabriz University of Medical Sciences, Tabriz, Iran

**Keywords:** Dislocation, Mandibular condyle, Osteotomy

## Abstract

Condylar dislocation is not an uncommon condition and occurs when the condyles are displaced anterior to the articular eminence and are unable to reduce back into the glenoid fossa. Long-standing dislocations are difficult to treat with the conservative methods and usually need surgical intervention. In this paper, a long-standing dislocation treated by bilateral extra-oral ramus osteotomy is described and the literature is reviewed.

## Introduction

Condylar dislocation (CD) is a type of hyper-mobile joint disorder and refers to an anteriorly displaced condyle which is not voluntarily reducible.^1^ Yawing or opening widely is a major problem in patients with joint hypermobility.^1^ Dislocation can also occur during bronchoscopy or laryngoscopy for the induction of general anesthesia.^2^ CD has been classified into acute, recurrent and chronic forms. Acute CD is the most common form and may develop as a result of congenital joint weakness, trauma, drug effects (phenothiazines), iatrogenic manipulation, and neurologic disorders.^1-4^

Long-standing condylar dislocation (LCD) occurs when the condyles are lodged anterior to the eminence for a period of more than 3 weeks. The most common cause of CD is failure to diagnose an acutely displaced or inappropriately treated condyle. Acute dislocation is usually managed by conservative methods such as manual reduction under local anesthesia with or without intravenous sedation with benzodiazepines and muscle relaxants, controlled tractions by intermaxillary elastics, and rarely under general anesthesia.^1-4^

On the other hand, the management of LCD is more complicated because of the masticatory muscle spasm and fibrous tissue formation at the joint area. Although some cases of prolonged dislocation have been treated by manipulative procedures,^5-8^ LCD may require surgical interventions such as condylectomy,^4^ eminectomy,^5^ and osteotomies.^6,9^ Debnath et al^6^ used an external approach for bilateral vertical oblique osteotomy of ramus in a case of long-standing bilateral dislocation of the temporomandibular joint where conventional methods were unsuccessful for reducing the dislocated condyle. In this paper, a case of LCD treated by extra-oral vertical osteotomy of the mandible is reported.

## Case Report 

**Figure 1.**
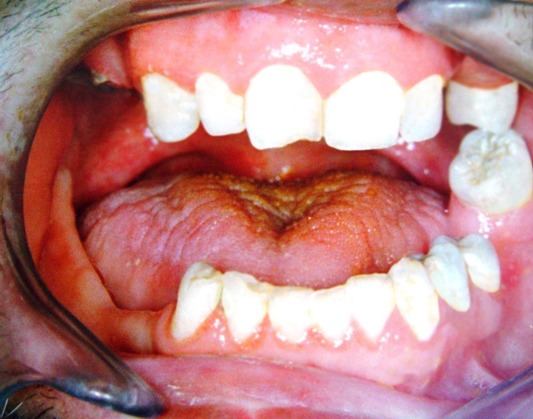
Intraoral view shows anterior open bite.

**Figure 2.**
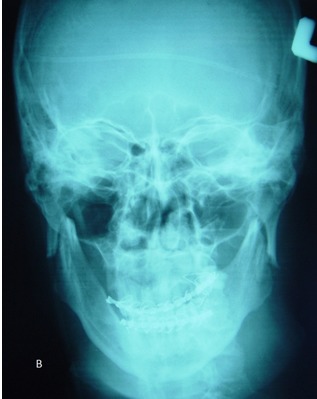
Panoramic view shows anterior displacement of the condyles.

**Figure 3.**
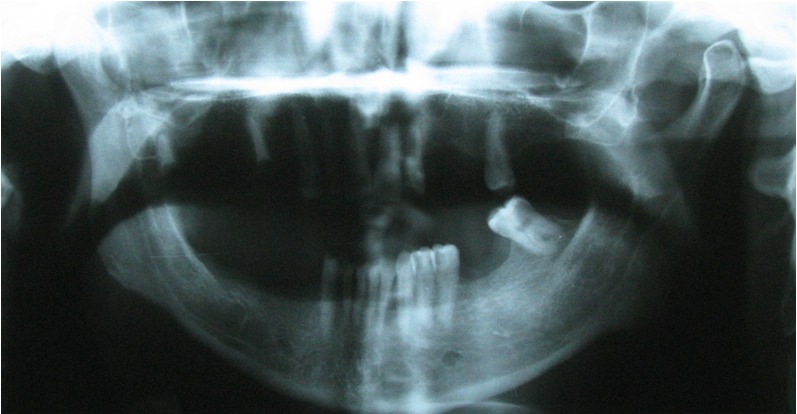
Traction wire passed through bur holes at the left mandibular angle.

A 40-year-old male was referred to the Department of Oral and Maxillofacial Surgery five months after a severe motor vehicle accident for treatment of pain and inability in closing the mouth. The patient had sustained severe head injury for which he had been hospitalized in the trauma department for 45 days. Craniotomy had been carried out and a cerebral shunt inserted to reduce intracranial pressure. In extra-oral examination, a deviation was present in the orbital axis, which was a sequel of the head injury. The mouth was found open and the mandible was anteriorly displaced (Figure 1). Depressions anterior to the tragus were visible bilaterally and the condyles were fixed and palpable in front of the ears. Intraoral findings included loss of several teeth; anterior displacement of the lower jaw, and anterior open bite with an inter-incisal distance of 40 mm. Anterior displacement of both condyles was seen on the panoramic radiograph (Figure 2) and confirmed by CT views. According to the findings from the history and physical examination, a diagnosis of irreducible long-standing condylar dislocation was established and the patient was scheduled for treatment under general anesthesia. The occlusal models were prepared to check the postoperative occlusion. The procedures were explained to the patient and a detailed consent form was obtained. General anesthesia was administered via naso-endotracheal tube using intravenous midazolam, phentanyl, muscle relaxants, and propofol. Under general anesthesia, noninvasive methods such as different modalities of jaw manipulation and controlled tractions failed to reduce the jaw. Subsequently, the angles of mandible on either side\were exposed via bilateral Risdon approaches to apply traction using bone hooks in the sigmoid notch and heavy traction wires passed through bur holes at the angle (Figure 3). These procedures also failed to bring the condyles into their appropriate position. Therefore, bilateral vertical osteotomies were carried out and the mandible was guided to the normal occlusion. Intermaxillary fixation was applied with arch bars after removing the throat pack and kept for 10 days; subsequently, active mouth opening exercises were ordered. Proximal and distal segments were checked and the incisions were closed in three layers.

**Figure 4.**
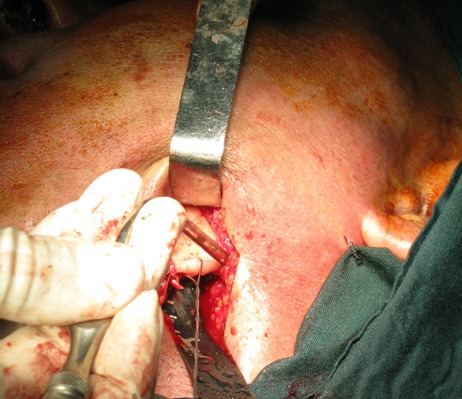
Postoperative view one year after surgery shows acceptable occlusion.

**Figure 5.**
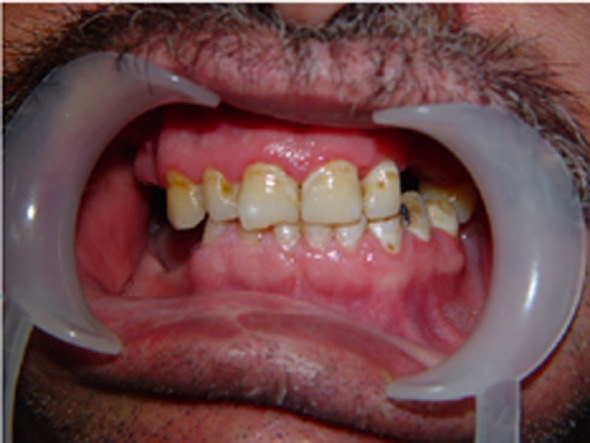
Postoperative panoramic view of the mandible shows relationship between the bone segments.

**Figure 6.**
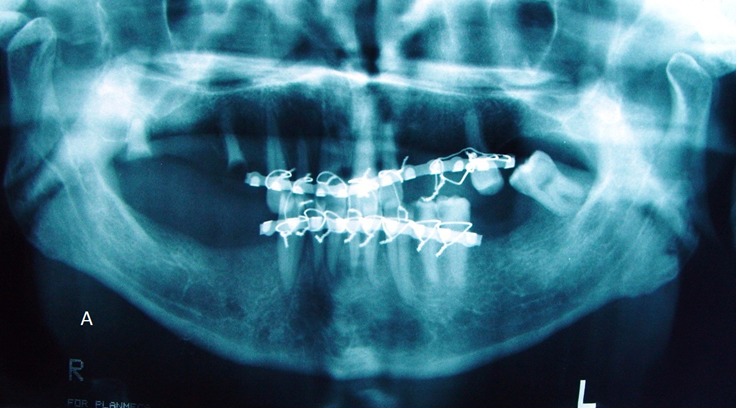
Postoperative PA view of the mandible shows relationship between the bone segments.

The postoperative period was uneventful and the patient was discharged from the hospital four days after surgery. The patient was followed regularly every week for the first month and then every 3 months. One year after surgery, the patient had normal occlusion with an inter-incisal opening of 40 mm without open bite (Figure 4). Despite the extra-glenoid position of the condyles on the postoperative panoramic view, the proximal segments revealed a good relationship with distal segments (Figure 5 &  6).

## Discussion

Once the dislocation of the TMJ has been established, attempts on reduction should be made as soon as possible. Local anesthetic block of auriculo-temporal nerve and lateral pterygoid muscle makes manipulations more comfortable for the patient.^1,2,4^Young et al^1^ treated a patient with an acute unilateral dislocation using masseteric and deep temporal nerve blocks. The condition was resistant to simple manipulation because of the elevator muscle spasm and severe pain. Although manual reduction of a dislocated condyle is the treatment of choice,^2,4,7^it was not possible in the presented case due to the long time interval between the injury and the treatment. Injection of the local anesthetics and intravenous midazolam were also unsuccessful. If mandibular dislocation is not treated, the condyles would be remained outside the glenoid fossa causing prolonged muscle spasm and fibrous tissue ingrowth inside the joint.^8^ This condition seems to be more prevalent in multi-trauma patients with long-term hospitalization and also edentulous patients wearing complete dentures for many years. In the presented case, early detection of the dislocation was hindered because of prolonged hospitalization in the intensive care unit for severe head injury. Several techniques have been described for the reduction of chronically displaced condyle. These techniques can be classified as non-surgical and surgical.^9^In non-surgical techniques, treatment may be achieved by manual reduction assisted by the use of muscle relaxants, local and general anesthesia or slow controlled traction by intermaxillary elastics. More invasive treatment modalities, including posterior traction by bone hooks or mandibular angle transosseous wires, replacement of condyle in the glenoid fossa, condylectomy or mandibular osteotomy procedures, should be considered in the case of conservative treatments failure.^1,5-9^ Lello^9^ used simultaneous traction on three zygomatic bone hooks to rotate the mandible about the mouth probe fulcrum. Blind insertion of three transcutaneous bone hooks is very traumatic, requiring a huge force to guide the mandible into the desired position, which might fracture the jaw or threaten vital structures. Treatments such as eminectomy or use of miniplates and screws in the articular eminence are usually used when the condyle head is reducible.^5,10^ The former treatment permits free movement of condyles and the latter limits the forward extrusion. Minzuno et al^5^ treated two patients with LCD using eminectomy.

Although condylectomy has been successfully used to achieve a satisfactory occlusal relationship in the treatment of non-reducible LCD,^4,7,8^ it is not always successful in positioning the mandible properly. This technique sometimes needs to be supported by coronoidectomy and supra-hyoid myotomy.^7^ Bilateral mandibular ramus osteotomies have successfully been used to treat LCD.^6^ Leaving the condyles in the dislocated position, these methods can solve the problem if too much dissection or trauma is required to free the condyles.^6^ Vertical ramus osteotomy is technically easier and can be carried out extra- or intra-orally. Despite the extra-glenoid position of condyles, the function of the joint is good and the relationship between the proximal and distal segments is acceptable. It seems that a new articulation established in front of the previous joint serves as a pseudo-joint which can maintain the function of TMJ properly. The surgical procedure is more comfortable and has fewer complications in comparison with other procedures using preauricular incisions for condylectomy or eminectomy.^6^ Moreover, attempts for repositioning the condylar head to a postero-superior location is not always possible because of fibrosis and scar formation in the joint capsule between the disk, condyle and articular eminence.^6,8^ In the present case, an extra-oral approach was selected to make it possible for the wires to pass through the holes placed in mandibular angles. If the patient is primarily a candidate for osteotomy, the intraoral approach is the treatment of choice. Subsequent to osteotomy, the patient should be placed in short-term intermaxillary fixation (10-14 days) to establish and maintain vertical dimension and prevent mandibular retrusion.

Judicious mouth opening exercises are an essential part of the treatment to maintain normal jaw function. The main objective of any treatment in LCD is to achieve a normal jaw relation with a normal range of motion.^5^ However, the difficulty encountered in treating mandibular dislocation increases with the duration of dislocation.^8^ With regard to these factors, it can be concluded that bilateral vertical ramus osteotomies for malocclusion correction can be the first treatment of choice if too much dissection and destruction of tissue is required to reposition the condyles.
